# Continuous Purification of mRNA Produced by In Vitro Transcription Using High Performance Countercurrent Membrane Purification

**DOI:** 10.1002/bit.70259

**Published:** 2026-06-03

**Authors:** Ziqiao Wang, Amin Javidanbardan, Ali Behboudi, Andrew L. Zydney

**Affiliations:** ^1^ Robert V. Waltemeyer Department of Chemical Engineering, The Pennsylvania State University University Park PA USA

**Keywords:** continuous processing, membrane purification, mRNA, ultrafiltration

## Abstract

Messenger ribonucleic acid (mRNA) therapeutics produced by in vitro transcription must be purified to remove residual enzymes and free nucleotides. This study examines the use of high‐performance countercurrent membrane purification (HPCMP) for the purification of mRNA therapeutics based on differences in the rate of diffusion across semipermeable polyethersulfone hollow fiber membranes. Experiments were performed using two model mRNA constructs using cytosine triphosphates (CTPs) and Proteinase K as model impurities. HPCMP achieved 99.7% removal of CTPs using a residence time of 60 min with no measurable loss of mRNA. Proteinase K removal by HPCMP was reduced in the presence of mRNA due to complexation between the positively charged protein and the negatively charged mRNA, an effect that was independently confirmed by dynamic light scattering and stirred cell diafiltration experiments. The HPCMP process could be operated continuously for more than 24 h without membrane fouling with stable mRNA yield (> 97%) and Proteinase K removal (> 94%). These results clearly demonstrate the feasibility of using HPCMP for continuous purification of mRNA therapeutics.

## 1 Introduction

Messenger Ribonucleic acid (mRNA) is a relatively new class of therapeutic, showing great promise due to its clinical versatility and rapid development timelines. mRNA‐based therapeutics are cost‐effective, simple to manufacture, and can target a broad range of diseases, including pathogenic infections as well as numerous metabolic, genetic, and cardiovascular disorders (Qin et al. 2022; Alberer et al. 2017; Feldman et al. 2019). mRNA‐based therapeutics are produced by in vitro transcription (IVT), where an RNA polymerase assembles the target mRNA from a reaction mixture containing the nucleotide triphosphates (NTPs) based on the sequence encoded in a linearized DNA template. The reaction product (the IVT crude) contains the desired mRNA along with residual nucleotides, the DNA template, the RNA polymerase, and possibly a range of product‐related impurities (double‐stranded RNA and truncated constructs), all of which must be removed by the downstream purification process to ensure the safety, stability, and efficacy of the final product (Lenk et al. 2024).

Membrane technology plays an essential role in the purification of mRNA‐based therapeutics. Current downstream processes for mRNA include multiple membrane‐based unit operations for concentration, removal of low molecular weight impurities, and buffer exchange, as well as sterile filtration of the final product (Javidanbardan et al. 2024). Conventional membrane‐based unit operations are performed in batch mode, where the feed is continuously recirculated through a tangential flow filtration module, with the desired concentration and/or purification factor achieved at the end of the process (Geiger and Treml 2020; Scorza et al. 2021; Funkner et al. 2016). These batch operations lead to broad residence time distributions and extended exposure to shear and pumping, potentially reducing product quality and yield.

There is increasing interest in the development of continuous and intensified bioprocessing operations, which can reduce capital equipment costs and facility size, increase productivity, provide greater manufacturing flexibility, and improve product quality (Zydney 2021). Royo et al. (2026) have specifically examined the potential benefits of developing an integrated continuous cGMP platform for mRNA manufacturing, including the ability to easily scale‐up/scale‐out the continuous platform to meet variability in demand, which is particularly important for the production of mRNA vaccines.

A number of recent studies have examined the development of continuous processes that could be incorporated in mRNA manufacturing. Javidanbardan et al. (2025) demonstrated the successful application of single‐pass tangential flow filtration (SPTFF) for continuous concentration of mRNA directly in the IVT crude mixture, although this provided relatively little purification. Royo et al. (2025) presented a continuous process for mRNA purification that uses a combination of precipitation and tangential flow filtration. This process minimized the need for sample preconditioning, but it required the use of significant quantities of polyethylene glycol that would need to be removed in subsequent steps in the overall process. Shahab et al. (2025) described the development of digital twin software for end‐to‐end continuous mRNA manufacturing using TFF, followed by continuous chromatography, lipid nanoparticle formulation, and freeze‐drying, although the application of continuous chromatography for mRNA purification is challenging.

Countercurrent membrane contacting could potentially provide a very attractive approach for continuous mRNA processing due to the very low shear operation, the absence of any preconditioning, and the ability to directly process the IVT crude (without the need for additives as in continuous precipitation). Countercurrent dialysis has been used for more than half a century in the treatment of chronic kidney disease (Bowry 2002), with hollow fiber membranes providing as much as 90% single‐pass removal of urea from blood with significant removal of lower molecular weight proteins such as β2‐microglobulin (with a MW ≈ 12 kDa) (Bhimani et al. 2010; Floge et al. 2010; Liao et al. 2003). More recently, Yehl et al. (2019) demonstrated that countercurrent dialysis can be effectively used for buffer exchange in the continuous processing of biopharmaceutical products. A similar approach can also be used for protein separation through a process known as high‐performance countercurrent membrane purification (HPCMP), achieving high‐resolution separation of myoglobin (MW = 17 kDa) and bovine serum albumin (67 kDa) by exploiting highly selective diffusive transport through the semipermeable membrane (Yehl and Zydney 2021). Mohammadzadehmarandi et al. (2023) subsequently showed that HPCMP can remove host cell proteins (HCP) derived from Chinese Hamster Ovary cells in the purification of a monoclonal antibody product, with > 95% HCP removal and 95% antibody yield. The operation of these systems without any significant pressure‐driven filtration significantly reduced membrane fouling, enabling long‐term continuous operation without replacing or cleaning the membrane. HPCMP has also been shown to reduce buffer consumption compared to both traditional batch diafiltration and affinity chromatography (Mohammadzadehmarandi et al. 2023).

The objective of this study was to examine the potential of using HPCMP for continuous purification and buffer exchange for mRNA therapeutics. Experiments were performed with commercially available modified polyethersulfone (mPES) hollow fiber ultrafiltration membranes with a 100 kDa nominal molecular weight cutoff (MWCO). Data were obtained with cytosine triphosphates to identify an appropriate range of operating conditions, as well as with a binary mixture of Proteinase K (a model enzyme) and purified mRNA to explore the effectiveness of the separation process. The results clearly demonstrate that HPCMP can achieve high degrees of both small solute and enzyme removal in the downstream processing of mRNA.

## 2 Materials and Methods

### 2.1 Materials

Two mRNA constructs were used: one that codes for firefly luciferase (Fluc with 2,117 nucleotides) and one for a self‐amplifying RNA (saRNA with 9,442 nucleotides). Both mRNA constructs were produced and purified by Arranta Bio (Boxborough, MA). The purified mRNA was supplied in water for injection and stored at −80°C until use.

HPCMP was performed by mixing the purified mRNA with either cytosine triphosphate (Hongene, Union City, CA) or Proteinase K (New England Biolabs), which was used as a model enzyme impurity. Proteinase K is a proteolytic enzyme that is used by Pfizer/BioNTech to degrade T7 polymerase and DNase I in the production of the Comirnaty vaccine (Patel et al. 2023).

### 2.2 High Performance Countercurrent Membrane Purification

HPCMP was performed using modified polyethersulfone (mPES) hollow fiber membranes with a nominal molecular weight cutoff of 100 kDa (Repligen Corp., Waltham, MA). The membranes were housed in MicroKros modules containing 6 fibers, each with an inner diameter of 0.5 mm and an active length of 20 cm, giving an effective membrane area of A = 20 cm^2^. Modules were operated with the feed (lumen inlet) flowrate controlled by a KDS Legato 210 syringe pump. A Masterflex L/S peristatic pump (Cole‐Palmer, Vernon Hills, IL), fitted with a single pump head having two separate channels, was used to control both the inlet and outlet flowrates for the draw solution (shell side). Pressure sensors (ESI technology, UK, GD4200‐USB) were located just before/after the inlet and outlet ports. Before each experiment, the module was flushed with deionized water followed by 40 mM Tris buffer at pH 7.4 using at least 20 L/m^2^. The feed reservoir was then replaced with the desired feed solution. Samples were collected periodically from the retentate and draw solution exits for offline analysis. At the end of the HPCMP experiment, the modules were flushed with water, cleaned using 0.5 M NaOH for a minimum of 30 min at 35°C–40°C, and stored in 0.1 M NaOH at 4°C for subsequent use.

### 2.3 Constant Flux Diafiltration

Constant flux diafiltration experiments were performed in an Amicon 8010 stirred cell (MilliporeSigma, Billerica, MA) with a feed volume of 10 mL. Small discs (4.1 cm^2^ membrane area) of 100 kDa Biomax polyethersulfone membrane were placed in the base of the stirred cell with an O‐ring used to prevent leakage. Membranes were flushed using at least 250 L/m^2^ of DI water, followed by approximately 20 mL of Tris buffer (pH 7.4). The filtrate port was then clamped, the buffer solution was replaced by the desired feed solution, the stirrer was set to 600 rpm, and the inlet port to the stirred cell was connected to a 60 mL Legato 210 syringe pump (KD Scientific) filled with 40 mM Tris buffer (pH 7.4). Filtrate samples were collected periodically for offline analysis.

### 2.4 Analytical Methods

Nucleotide concentrations were determined by size exclusion chromatography (SEC) using an Agilent Infinity 1260 Bio‐SEC system equipped with an Agilent Bio‐SEC column packed with 3 µm silica particles having a nominal pore size of 100 Å. 40 mM Tris buffer (pH 7.4) was used as the mobile phase at a flowrate of 0.35 mL/min. The column temperature was maintained at 25°C. Concentrations were determined by UV absorbance using calibration curves constructed by serial dilution of the stock solution.

mRNA transmission and integrity were evaluated by Ion Pair Reverse Phase (IP‐RP) high‐performance liquid chromatography performed with an Agilent Infinity 1260 system using a PLRP‐S 4000 Å column having a poly(styrene divinylbenzene) stationary phase (Agilent). Samples were analyzed by gradient elution using buffer A (0.1 M TEAA) and buffer B (0.1 M TEAA 25% v/v Acetonitrile) with the following conditions: 10% B for 5 min, 10% B to 75% B over 30 min, and then 100% B for 5 min, all at a flowrate of 1 mL/min (Azarani 2001). Detection was by UV absorbance at 260 nm, with concentrations determined by integration of peak areas.

Proteinase K concentrations in the presence of mRNA were evaluated by cation exchange chromatography using an SP Sepharose Fast Flow (Cytiva) resin having a 6% crosslinked agarose stationary phase with sulfopropyl charge groups. Elution was performed using buffer A (50 mM MES pH 6) and buffer B (50 mM MES 1 M NaCl pH 6) with the following conditions: 0% B for 5 min, 30% B for 10 min, 100% B for 5 min, and then 0% B for 10 min, all at a flowrate of 1 mL/min. Detection was by UV absorbance at 280 nm, with concentrations determined by comparison of the peak areas using a calibration curve constructed by serial dilution of the stock solution.

Protein‐mRNA interactions were also examined by dynamic light scattering using a Malvern Zetasizer Nano ZS90 (Malvern, UK). The sample was illuminated with a 632.8 nm laser, with the 90° scattered light intensity evaluated using an avalanche photodiode detector. Ten repeat measurements were obtained, each with a 30 s acquisition time. The diffusion coefficient was calculated from the measured correlogram using a non‐negatively constrained least squares fitting algorithm with a refractive index of 1.33 for the 40 mM Tris buffer.

## 3 Results and Discussion

### 3.1 NTP Removal by HPCMP

Initial HPCMP experiments were performed to determine the range of flowrates capable of providing a high degree of NTP removal. Data were obtained with a feed containing a 2 mM solution of cytosine triphosphate (CTP) in 40 mM Tris buffer at pH 7.2; the 2 mM nucleotide concentration is consistent with literature results showing initial NTP concentrations of 5–10 mM with NTP conversions of 50%–80% (Stover et al. 2026). Experiments were performed with the ratio of the draw solution to feed flowrate maintained at *α* = q_D_/q_F_ = 4 to provide a significant concentration driving force across the membrane (Yehl and Zydney 2022). The fractional CTP removal was evaluated as:

(1) $$R=1-\frac{q_{r}C_{r}}{q_{F}C_{F}}$$

where *q*_*r*_ and *C*_*r*_ are the flowrate and CTP concentration in the retentate exit, respectively. Mass balance closure was very good under all conditions (within ±5%), with the exit retentate flowrate within ±1% of the feed flowrate. Data were obtained with a single module, with the system allowed to equilibrate for a minimum of 6 h at each flow rate before evaluating the CTP removal.

**Figure 1:**
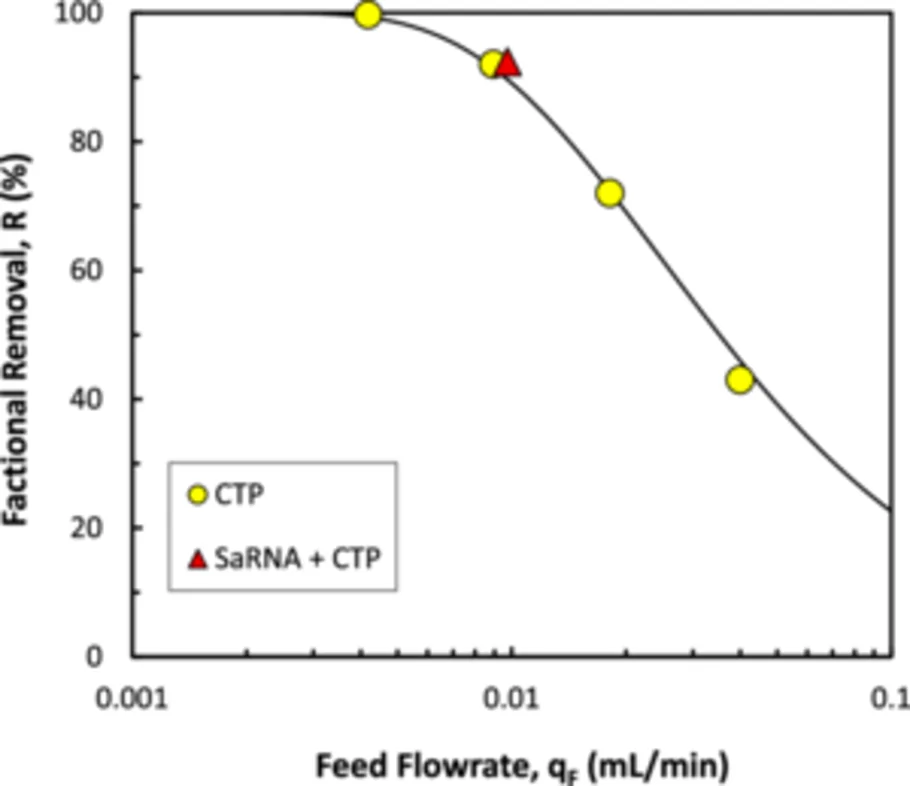
Effect of feed flowrate on the fractional removal of cytosine triphosphate for experiments performed with the MicroKros hollow fiber module with 100 kDa molecular weight cutoff membranes using a feed with 2 mM CTP and a ratio of draw to feed solution flow rates of α = 4. Symbols represent the average of 4 consecutive measurements; 95% confidence intervals are within the size of the symbols. The solid curve is the model calculation given by Equation (2).

The fractional CTP removal is shown as a function of the feed flowrate in Figure 1. In each case, the fractional removal was independent of time (over a period of 16 h at a given flow rate), with the reported values representing the average of the last 4 data points, all obtained after equilibration. The fractional CTP removal decreases with increasing flow rate due to the reduction in residence time within the hollow fiber module. Note that q_F_ = 0.01 mL/min corresponds to a residence time of 24 min based on the 0.24 mL volume of the hollow fibers (neglecting any hold‐up volume in the inlet or outlet headers). HPCMP was able to achieve 99.7% CTP removal at a feed flow rate of 4 µL/min (residence time of 60 min).

The solid curve in Figure 1 is the calculated degree of CTP removal in a continuous countercurrent contactor (Eloot 2004):

(2) $$R=1-\frac{\alpha -1}{\alpha \mathrm{Exp}\left[\beta \right]-1}$$

where β provides a measure of the mass transfer efficiency:

(3) $$\beta =\frac{k_{o}A}{q_{d}}\left(1-\frac{1}{\alpha }\right)$$

The best fit value of k_o_, the overall mass transfer coefficient for CTP, was determined by minimizing the sum of the squared residuals between the model and experimental data, giving k_o_ = 2.2 ± 0.1 × 10^−7 ^m/s for the data in Figure 1. The model calculations are in very good agreement with the experimental data over the full range of feed flow rates (with *R* ^2^ > 0.99). The sum of the squared residuals, defined as

(4) $$E=\sum {{(R}_{\text{exp}}-R_{\mathrm{model}})}^{2}$$

where, R_exp_ and R_model_ are the values of the fractional CTP removal determined experimentally and from the model, was E = 7.4 × 10^−4^, corresponding to a root mean squared error of 1.4%. The model thus provides a simple approach to calculate the operating conditions required to achieve any targeted degree of CTP removal.

Also shown in Figure 1 is a single data point obtained using a binary mixture with 0.2 mg/mL of purified saRNA added to the 2 mM CTP solution. The fractional CTP removal in this experiment, performed at q_F_ = 0.01 mL/min, was *R* = 92 ± 2% compared to *R* = 89% as given by Equations (2) and (3) under the same conditions. Thus, there is no evidence for any interactions between the saRNA and the CTP, which is consistent with the significant negative charge on both the free nucleotide and the large mRNA. These data also suggest that there is negligible fouling of the membrane by the saRNA, with this low degree of membrane fouling likely arising from the lack of any net pressure‐driven filtration in the HPCMP experiment.

### 3.2 Proteinase K Removal by HPCMP

**Figure 2:**
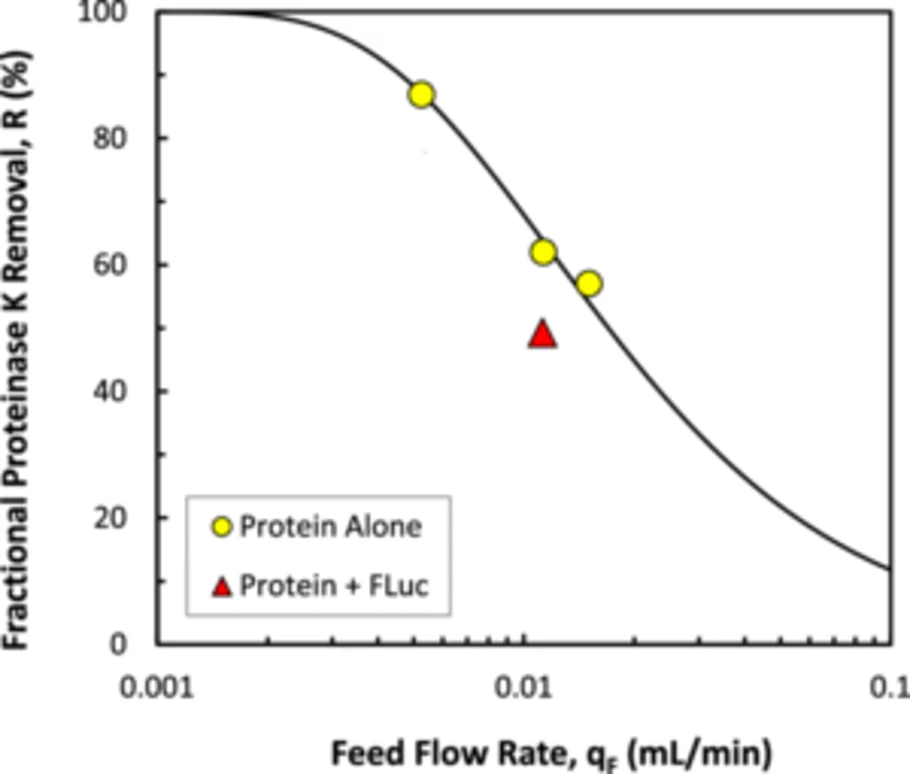
Effect of feed flow rate on fractional removal of Proteinase K for experiments performed with the MicroKros hollow fiber module with 100 kDa molecular weight cutoff membranes using a feed with 0.2 mg/mL Proteinase K and a ratio of draw to feed solution flow rates of α = 4. The solid curve is the model calculation given by Equations (2) and (3). Filled triangle is in the presence of 0.2 mg/mL Fluc mRNA.

Figure 2 shows a corresponding series of HPCMP experiments performed using Proteinase K at a concentration of 0.2 mg/mL. This is slightly higher than the 0.05–0.1 mg/mL concentration range usually recommended for Proteinase K (Beckert and Masquida 2011; Chillón et al. 2015), although Proteinase K concentrations as high as 1 mg/mL have been used for protein degradation in the extraction of nucleic acids from tissue samples (Proteinase K 2026). The larger Proteinase K concentration made it easier to detect the low concentrations in the draw solution. The solid curve is determined from Equations (2) and (3) using the best fit value of k_o_ = 1.1 ± 0.1 × 10^−7 ^m/s for the Proteinase K. The model is again in excellent agreement with the data (*R* ^2^ > 0.98), properly capturing the reduction in Proteinase K removal with increasing feed flowrate. The sum of the squared residuals was E = 0.0011, corresponding to a root mean squared error of 2%. However, in contrast to the data with CTP, the experiment performed in the presence of 0.2 mg/mL of the Fluc mRNA gave a fractional proteinase K removal of *R* = 49%, which was significantly smaller than that for the protein alone (*R* = 62%) and for the model (*R* = 63%), all at q_F_ = 0.011 mL/min. This behavior is discussed in more detail subsequently.

### 3.3 Mass Transfer Analysis

For purely diffusive transport across a porous membrane, the mass transfer coefficient is given as:

(4) $$k_{m}=\frac{\epsilon D_{\infty }}{\delta_{m}\tau }$$

where D_∞_ is the free solution diffusivity and $\epsilon ,\delta_{m},\text{and}\;\tau$ are the membrane porosity, thickness, and tortuosity, respectively. Equation (4) gives k_m_ = 6.2 × 10^−7 ^m/s using a free solution diffusivity for CTP of D_∞ _= 5.0 × 10^−10^ m^2^/s with ϵ = 0.5, $\delta_{m}$ = 200 µm, and τ = 2 (all typical for hollow fiber ultrafiltration membranes). This value is nearly 3× that determined from the data in Figure 1, a difference that is difficult to explain based on the properties of the hollow fiber membrane. Instead, the lower value of k_o_ suggests that there are significant contributions to the overall mass transfer resistance arising from the external boundary layers in the fiber lumen and/or the shell region within the hollow fiber membrane.

The best fit value of k_o_ = 1.1 × 10^−7 ^m/s for the Proteinase K data in Figure 2 is only a factor of two smaller than that for the CTP, despite the sixfold difference in the bulk diffusion coefficients: D_∞_ = 0.83 × 10^−10^ m^2^/s for Proteinase K compared to D_∞ _= 5.0 × 10^−10^ m^2^/s for CTP. This could be due to electrostatic repulsion of the negatively charged CTP from the negatively charged pores in the size‐selective skin of the highly asymmetric polyethersulfone membrane, which would reduce the partition coefficient of the CTP into the membrane pores (Glandt 1981), although one would expect the skin to provide relatively little resistance to mass transfer given its small thickness (< 1 µm). Alternatively, previous studies of mass transfer in hollow fiber membrane modules have shown that the mass transfer coefficient in the shell region is nearly independent of the solute diffusion coefficient (Wickramasinghe et al. 1992). If the shell‐side boundary layer provides a significant resistance to mass transfer, this would not only explain the small value of the overall mass transfer coefficient for the CTP, it could also explain the weak dependence of k_o_ on the solute diffusion coefficient based on the results for the CTP and Proteinase K. Additional studies will be required to develop a more quantitative analysis of mass transfer in HPCMP.

### 3.4 Proteinase K–mRNA Interaction

**Figure 3:**
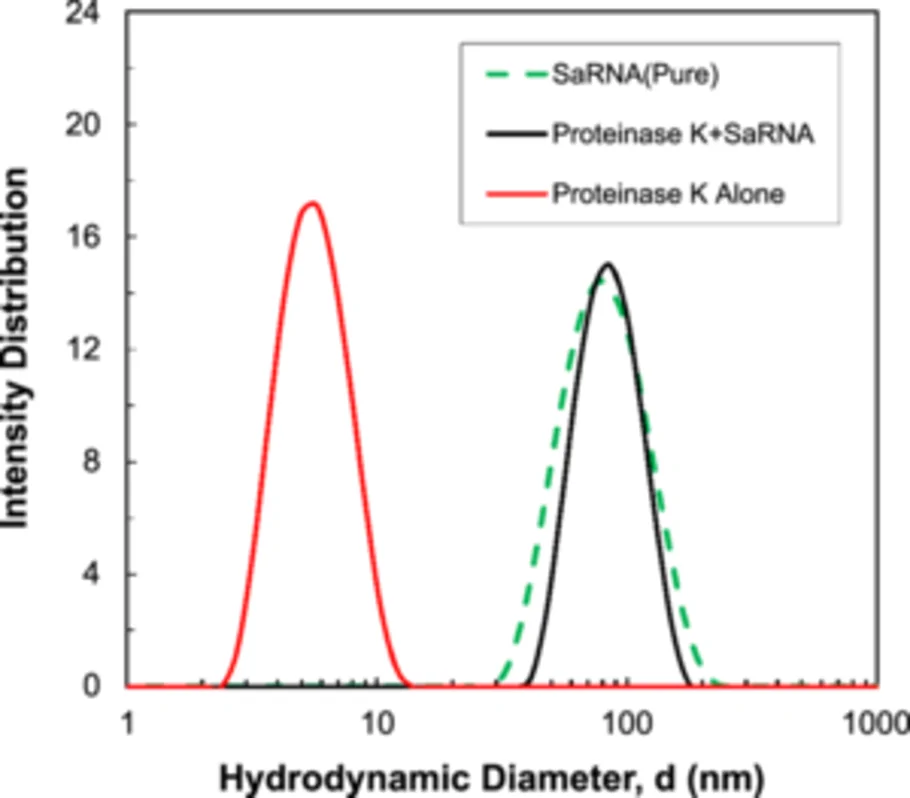
Light scattering intensity for Proteinase K alone (red), purified saRNA (dashed green), and a binary mixture of Proteinase K and saRNA (black). Data were obtained at an saRNA concentration of 0.5 mg/mL and a Proteinase K concentration of 6 mg/mL, both in 40 mM Tris buffer at pH 7.4.

Our hypothesis is that the lower fractional removal of Proteinase K in the presence of mRNA observed in Figure 2 is due to intermolecular interactions between the positively‐charged Proteinase K (isoelectric point of pI = 8.9) and the negatively‐charged mRNA. This was examined experimentally by dynamic light scattering (DLS), with results for the intensity‐weighted size distributions shown in Figure 3. Data obtained with the pure Proteinase K (at a concentration of 6 mg/mL) show a single peak with a mean diameter of approximately 6 nm, consistent with the 28.9 kDa molecular weight and with DLS data in the literature (Creese 2019). Corresponding results for the purified saRNA (at a concentration of 0.5 mg/mL) gave a single peak with a mean diameter of 73 nm; the use of the large saRNA in these experiments provided a sharp delineation between the peaks for the Proteinase K and saRNA. The intensity distribution for the binary mixture of Proteinase K and saRNA shows only a single peak with a slightly larger mean diameter than that of the saRNA alone, with no evidence of any free Proteinase K (although this is difficult to quantify due to the strong dependence of the scattering intensity on size). Note that parallel experiments performed with a negatively‐charged protein (bovine serum albumin) showed two distinct peaks in the binary mixture (data not shown), suggesting that the strong interactions between Proteinase K and the saRNA are primarily electrostatic in nature under the conditions of these experiments.

**Figure 4:**
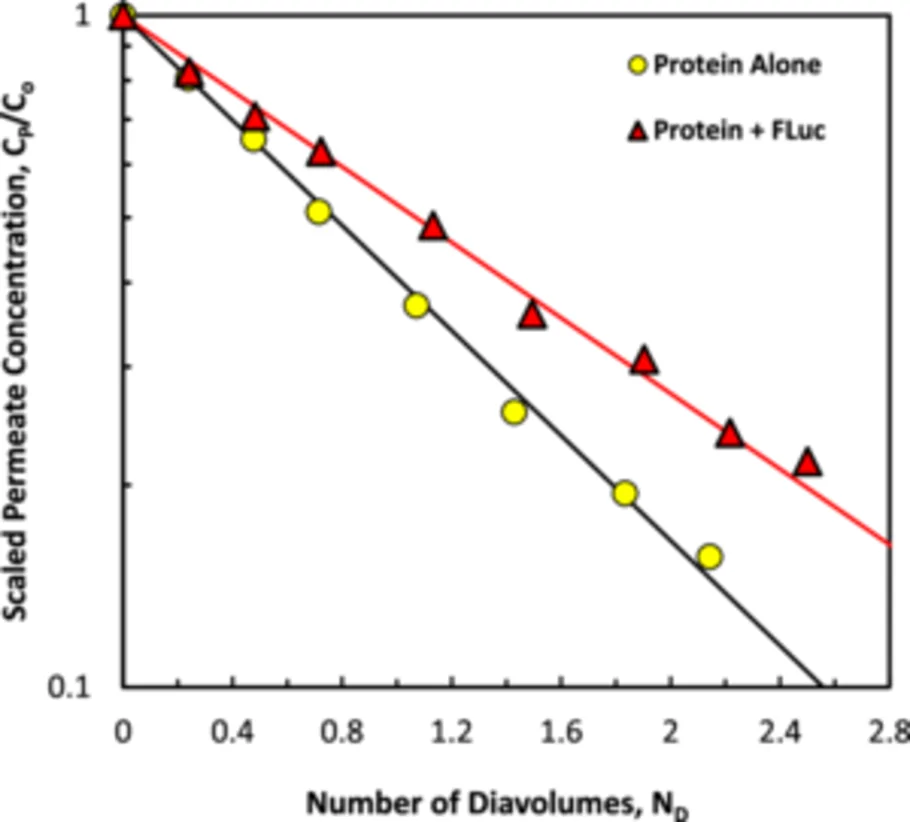
Scaled permeate concentration of Proteinase K during diafiltration in an Amicon 8010 stirred cell using a Biomax 100 kDa membrane at a filtrate flux of 60 L/m^2^/h. Solid lines are model calculations using Equation (5) with the best fit values of the observed sieving coefficients: S_0_ *=* 0.90 ± 0.03 for the proteinase K alone and S_o_ *=* 0.65 ± 0.02 for the proteinase K in the presence of mRNA.

Further confirmation of the interactions between Proteinase K and mRNA was obtained by performing diafiltration experiments in the stirred ultrafiltration cell. Figure 4 shows results for diafiltration of 0.2 mg/mL solutions of Proteinase K, both alone and in the presence of the Fluc mRNA at a concentration of 0.2 mg/mL. Corresponding experiments performed with the pure mRNA showed essentially complete retention of the mRNA, with no mRNA detected in any of the permeate samples (data not shown). Data were obtained at a constant permeate flux of 60 L/m^2^/h, with the transmembrane pressure remaining nearly constant at 20 kPa (3 psi) throughout the diafiltration. Results are shown for the scaled permeate concentration as a function of the number of diavolumes (N_D_), which is defined as the cumulative filtrate volume divided by the constant feed volume in the stirred cell. The results are highly linear on the semi‐log plot (*R* ^2^ > 0.995), in good agreement with the expected exponential decay during diafiltration (Van Reis and Saksena 1997):

(5) $$\frac{C_{p}}{C_{\mathrm{po}}}=\text{exp}\left(-N_{D}S_{o}\right)$$

where C_p_ is the Proteinase K concentration in the permeate solution during the diafiltration and C_po_ is the initial permeate concentration. The best fit values of S_o_, the observed sieving coefficient for Proteinase K, were obtained by simple linear regression fits, giving S_o_ = 0.90 ± 0.03 for the Proteinase K alone and S_o_ = 0.65 ± 0.02 for the Proteinase K in the presence of the mRNA. Since the Proteinase K‐mRNA complex should be fully retained by the 100 kDa membrane, these data suggest that approximately 30% of the Proteinase K is complexed with the mRNA and thus retained by the Biomax 100 kDa membrane.

### 3.5 Proteinase K Removal in Presence of mRNA

**Figure 5:**
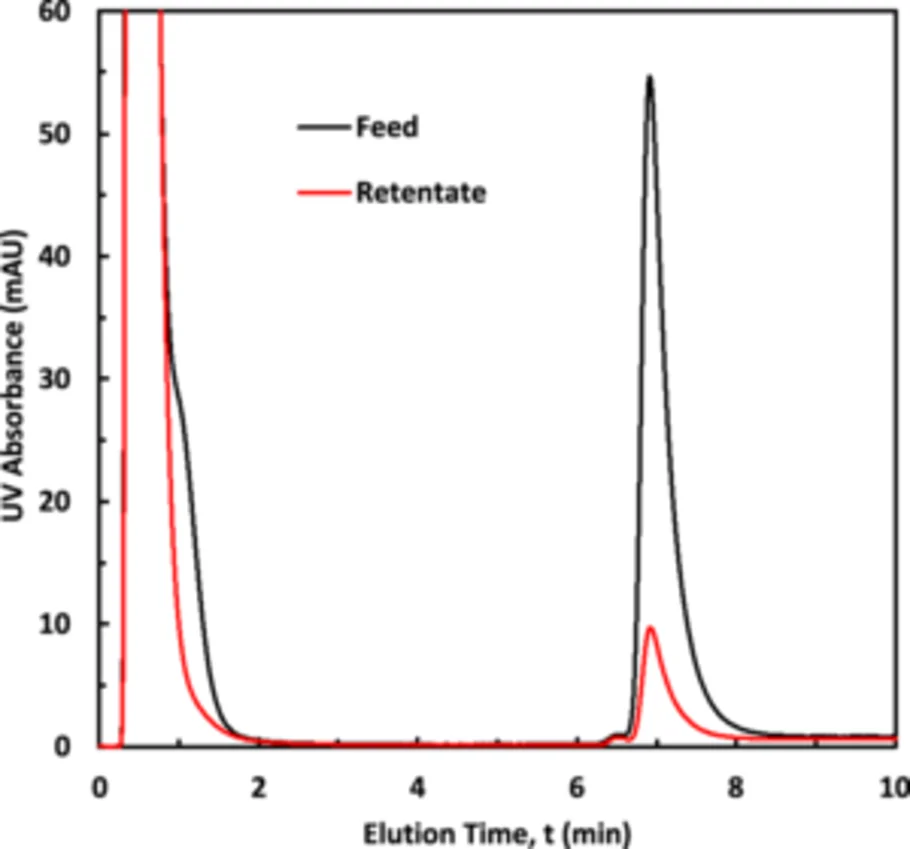
CEX chromatograms for the feed and retentate product from HPCMP performed at a feed flowrate of 0.005 mL/min and a draw solution flowrate of 0.02 mL/min for a feed containing mRNA and Proteinase K, both at concentrations of 0.2 mg/mL.

Figure 5 shows results from an HPCMP experiment performed with a binary mixture of Proteinase K and Fluc mRNA, both at a feed concentration of 0.2 mg/mL, with q_F_ = 0.005 mL/min and *α* = 4. The very large peak in the CEX chromatograms collected in the flow‐through (between 0.5 and 2 min) corresponds to the mRNA and any components that do not interact with the negatively charged resin. The tailing shoulder observed in the feed is likely due to additives that were present in the Proteinase K stock solution, such as glycerol; this shoulder was observed in both the feed and permeate samples. The peak that elutes between 6 and 8 min (at higher ionic strength) is the Proteinase K. The HPCMP process provides approximately 80% removal of Proteinase K under these conditions based on integration of the peak areas. Samples obtained from the draw solution exit showed no measurable mRNA, with good mass balance closure (within 5%) for the Proteinase K.

**Figure 6:**
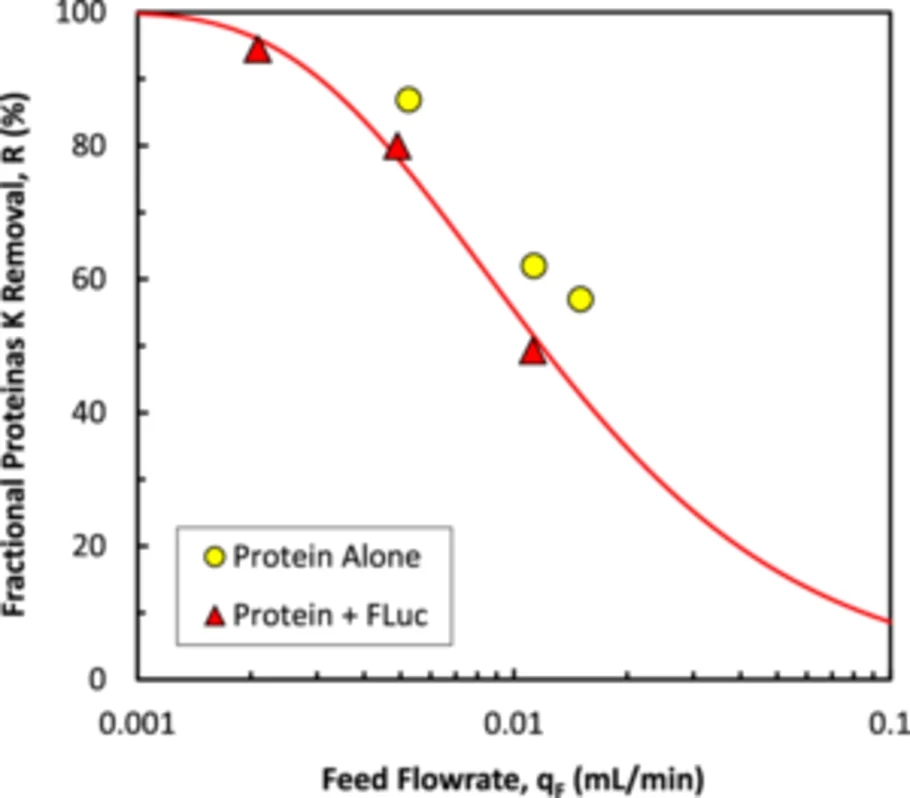
Effect of feed flowrate on fractional removal of Proteinase K, both alone and in the presence of Fluc mRNA. Solid curve is model prediction developed from Equation (7) with k_o_ *=* 1.1 × 10^−^^7^ m/s and *f* *=* 0.28.

The results from Figure 5 are replotted in Figure 6 as the fractional Proteinase K removal along with data for a series of HPCMP experiments performed over a range of feed flowrates, all with *α* = 4. Also shown for comparison are data obtained with the Proteinase K alone (results from Figure 2). The solid curve in Figure 6 is a model calculation accounting for partial (reversible) complexation of the Proteinase K with the mRNA, with only the free Proteinase K able to pass through the membrane:

(6) $$q_{R}\frac{dC_{R}}{\mathrm{dz}}=-k_{o}a\left[\left(1-f\right)C_{R}-C_{D}\right]$$

where q_R_ and C_R_ are the local Proteinase K concentrations in the retentate, a is the membrane area per unit length, and f is the fraction of Proteinase K that is complexed with the mRNA. Equation (6) can be combined with a corresponding mass balance in the draw solution and integrated over the length of the membrane to evaluate the overall fractional Proteinase K removal giving:

(7) $$R=1-\frac{\left(1-f\right)\alpha -1}{\left(1-f\right)\alpha \text{exp}\left[\beta \right]-1}$$

with α and β given by Equations (2) and (3), respectively. The overall mass transfer coefficient for Proteinase K was taken as k_o_ = 1.1 × 10^−7 ^m/s based on the results in Figure 2 for Proteinase K alone, with *f* = 0.28 determined from the results obtained in the diafiltration experiment:

(8) $$f=1-\frac{S_{o,\mathrm{mRNA}}}{S_{o,\mathrm{alone}}}$$

where S_o, mRNA_ is the observed sieving coefficient for Proteinase K in the presence of mRNA and S_o, alone_ is the observed sieving coefficient for Proteinase K alone. The model predictions are in excellent agreement with the experimental data (*R* ^2^ > 0.99) over the full range of flow rates, with E = 0.0013 (corresponding to a root mean squared error of 2.1%), demonstrating that the reduction in fractional Proteinase K removal in the presence of the mRNA is directly due to the association between Proteinase K and Fluc mRNA. The model predicts that more than 99% of the Proteinase K could be removed by using q_F_ = 0.0014 mL/min with α = 4, while the batch diafiltration data in Figure 4 suggest that 7 diavolumes would be required to achieve the same level of Proteinase K removal. Thus, the HPCMP process uses 43% less buffer than that required for a batch diafiltration, consistent with results presented in previous studies of HPCMP for antibody purification (Yehl and Zydney 2021; Mohammadzadehmarandi et al. 2023).

### 3.6 HPCMP Performance

**Figure 7:**
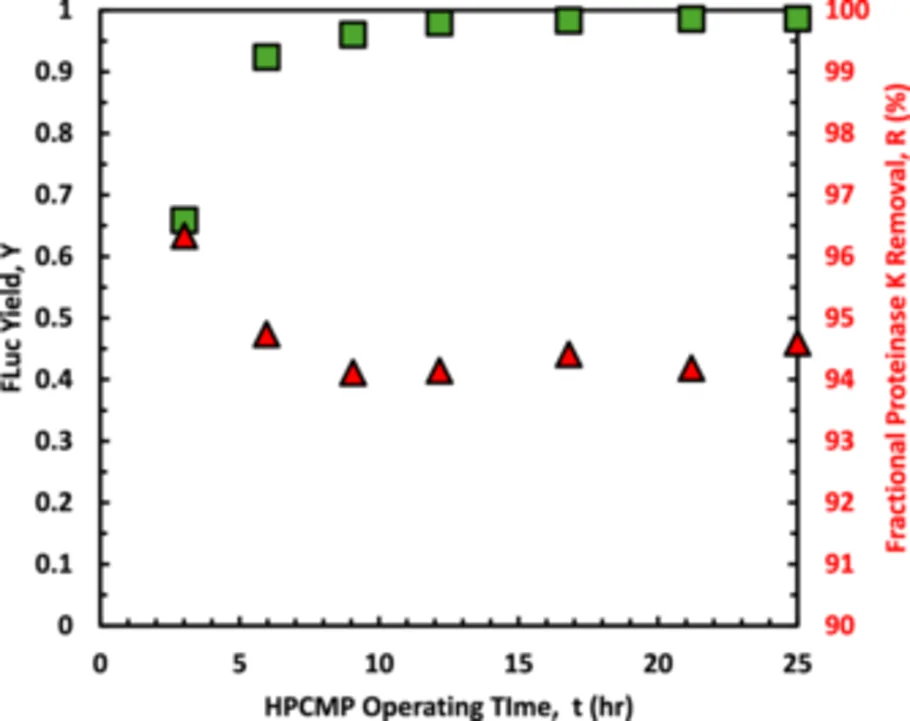
Fluc mRNA yield and fractional Proteinase K removal as a function of time during HPCMP. Data were obtained at a feed flowrate of 0.002 mL/min with *α* = 4 for a feed containing 0.2 mg/mL of the Fluc mRNA and Proteinase K.

One of the advantages of HPCMP is that it can be performed continuously at steady‐state with minimal degradation in membrane performance due to the negligible rates of membrane fouling. This was explored in Figure 7 for an HPCMP experiment performed in the MicroKros hollow fiber module at a feed flowrate of 0.002 mL/min with *α* = 4 using a binary mixture of Proteinase K and purified Fluc mRNA, both at a feed concentration of 0.2 mg/mL. The module and tubing were initially flushed with buffer, with the feed reservoir replaced with the mRNA/Proteinase K solution at *t* = 0. The difference between the retentate and feed flowrates was less than 3% throughout the experiment, with excellent mass balance closure (within ±5%).

Samples were obtained from the exit (retentate) solution over the 25 h experiment and analyzed by cation exchange chromatography. The proteinase K concentration at *t* = 3 h was 3.6% of the initial feed due to the combination of Proteinase K dilution by the holdup volume and Proteinase K removal by HPCMP. The fractional Proteinase K removal attains a nearly constant value after 6 h with greater than 94% Proteinase K removal throughout the 25 h experiment. The mRNA yield in the retentate product increased as the hold‐up volume was washed through the system, with the mRNA yield for the final 15 h of operation remaining above 98%.

**Figure 8:**
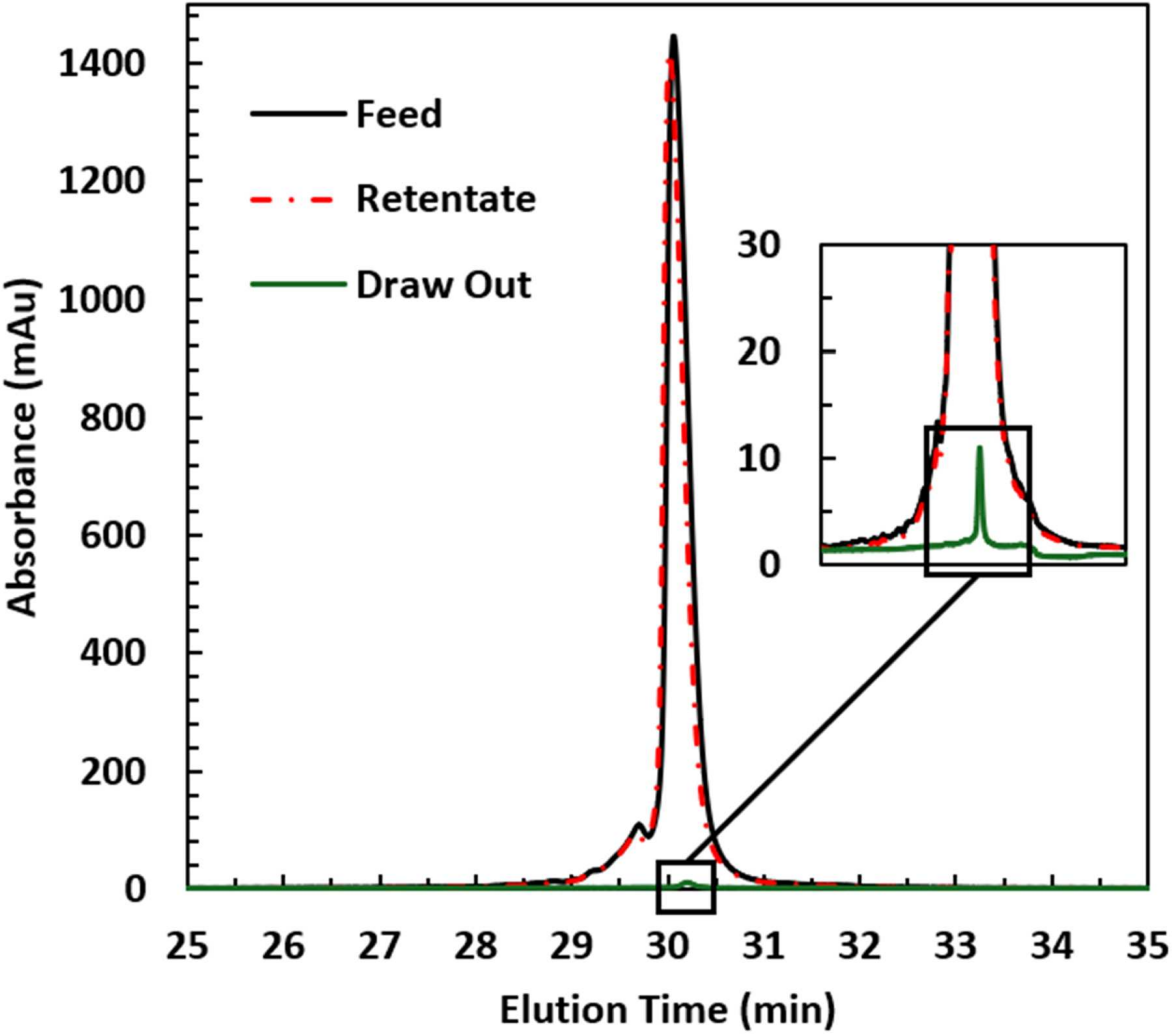
IP‐RP chromatograms for the feed, retentate, and draw outlet solutions from HPCMP performed at a feed flow rate of 0.002 mL/min and a draw solution flow rate of 0.008 mL/min for a feed containing mRNA and Proteinase K, both at concentrations of 0.2 mg/mL.

The integrity of the purified Fluc mRNA obtained from the HPCMP experiment was examined using Ion‐Pair Reverse‐Phase High Performance Liquid Chromatography, with results for samples obtained at *t* = 16.8 h for the experiment in Figure 7 shown in Figure 8. The mRNA peaks for the feed and exit (retentate) product were nearly identical, with no evidence of any mRNA degradation during the HPCMP process. In addition, the removal of the Proteinase K had no observable effect on the integrity of the mRNA, as expected. There was a very small mRNA peak visible in the collected draw solution (insert in Figure 8), corresponding to approximately 0.3% of the initial mRNA, consistent with the very small reduction in the height of the mRNA peak in the retentate sample compared to that in the feed solution.

## 4 Conclusions

The data presented in this study provide the first demonstration that high‐performance countercurrent membrane purification (HPCMP) can be used for the purification of messenger ribonucleic acid (mRNA) therapeutics. HPCMP was performed using commercially available 100 kDa nominal molecular weight cutoff modified polyethersulfone hollow fiber membranes, providing a high degree of removal of both cytosine triphosphate (CTP) and Proteinase K, a proteolytic enzyme that is used in the production of Comirnaty to degrade both DNase and residual T7 RNA Polymerase.

The effectiveness of impurity removal can be increased by increasing the residence time in the membrane module, i.e., by operating at a lower feed flowrate. Data for small nucleotide removal were in excellent agreement with predictions of classical models for countercurrent contacting. In contrast, Proteinase K removal was reduced in the presence of mRNA due to the formation of a reversible complex between the positively charged enzyme and the negatively charged mRNA. This interaction was independently confirmed by dynamic light scattering and quantified using constant flux diafiltration experiments. Incorporation of the binding fraction into the model for countercurrent contacting provided excellent agreement with experimental data over a wide range of operating conditions, providing a simple theoretical framework that can be used to identify operating conditions that provide the desired removal of both small impurities (e.g., free nucleotides) and residual enzymes (like Proteinase K).

HPCMP provides a single‐pass, continuous process under low shear conditions, with no observable degradation in the mRNA as determined by Ion Pair Reverse Phase chromatography. In addition, there was no loss in performance over more than 24 h of continuous operation, suggesting that membrane fouling is negligible in this diffusive process, consistent with previous results. However, it is important to note that the mRNA concentration used in this work (0.2 mg/mL) is lower than typical industrial titers (around 2–5 mg/mL); this lower concentration was used given the limited availability of purified mRNA. HPCMP performance should be largely independent of the mRNA concentration, although the higher concentration feed will provide a greater concentration driving force for diffusion. In addition, it will be important to examine the effect of other impurities in the IVT crude on the HPCMP process, including multivalent cations like spermine. Future studies will be required to evaluate the effects of mRNA concentration and impurity levels on the HPCMP process and to demonstrate the direct integration of HPCMP as part of a fully continuous process for mRNA manufacturing.

## Author Contributions

**Ziqiao Wang:** data curation, formal analysis, investigation, writing – original draft; **Amin Javidanbardan:** formal analysis, investigation, writing – review and editing; **Ali Behboudi:** formal analysis, investigation, writing – review and editing; **Andrew L. Zydney:** formal analysis, funding acquisition, investigation, supervision, writing – review and editing.

## Disclosure

This report was prepared as an account of work sponsored by an agency of the United States Government. Neither the United States Government nor any agency thereof, nor any of their employees, makes any warranty, express or implied, or assumes any legal liability or responsibility for the accuracy, completeness, or usefulness of any information, apparatus, product, or process disclosed, or represents that its use would not infringe privately owned rights. Reference herein to any specific commercial product, process, or service by trade name, trademark, manufacturer, or otherwise does not necessarily constitute or imply its endorsement, recommendation, or favoring by the United States Government or any agency thereof. The views and opinions of authors expressed herein do not necessarily state or reflect those of the United States Government or any agency thereof.

## Conflicts of Interest

The authors declare no conflicts of interest.

## Data Availability

The data that support the findings of this study are available from the corresponding author upon reasonable request.
